# On heights and plains: How rodents from different habitats cope with three-dimensional environments?

**DOI:** 10.1371/journal.pone.0265176

**Published:** 2022-03-10

**Authors:** Zohar Hagbi, David Eilam

**Affiliations:** George S. Wise Faculty of Life-Sciences, School of Zoology, Tel-Aviv University, Ramat-Aviv, Israel; Ben-Gurion University of the Negev, ISRAEL

## Abstract

Dwelling in a specific habitat requires adaptation to the habitat physical and biological properties in order to maximize fitness. Adaptations that are manifested in the organization of behavior in time and space reflect how the environment is perceived and utilized. Testing species from different habitats in the same laboratory environment can uncover the differences in their behavior and their adaptations to specific habitats. The question posed in this study is that of how two rodent species, one occupying flatlands (Tristram’s jird; *Meriones tristrami*) and the other occupying structured rocky habitats (common spiny mouse; *Acomys dimidiatus*), differ in the way that they explore the same three-dimensional laboratory environment. Individuals of these two species were introduced into an arena with a five-level ziggurat in the center, and their behavior was followed for 60 min. We found that both species preserved the typical spatiotemporal rodents’ behavior of establishing a home-base—a location that is a terminal from which they set out to explore the environment. However, the jirds, which live in flatlands, mainly travelled on the arena floor and the lower levels of the ziggurat; while, in contrast, the spiny mice, which live in rocky habitats and are used to climbing, mostly remained and travelled on the ziggurat, with some of them hardly descending to the arena floor. We suggest that the distinction in spatial behavior between the two species reflects their different motor abilities, different depth perception, and different *umvelt* (perceived world), in accordance with their different natural habitats.

## Introduction

Different species occupy different habitats that vary in their physical structure and biological properties, thereby providing different opportunities for living and foraging. In each habitat, animals need to balance among various factors, such as the cost of foraging, predation risk, intake of energy from food, chances of finding a mate etc., in order to optimize foraging and increase their fitness [1,2]. Accordingly, each habitat poses different challenges, requiring different motor abilities and sensory perceptions and conceptions of the specific environment. Such specialization is striking in the case of related species. For example, kittiwake gulls (*Larus rissa tridactyla*) that nest on cliffs differ in various ways from other related gull species that nest on the ground—herring gulls (*Larus argentatus*) and black-headed gulls (*Larus ridibundus*; [3]). Compared with the two latter related species, kittiwake gulls have adapted different nest shape and location, copulation behavior, clutch size, alarm call and response to predators, and even their chicks respond differently to attacks [3]. Dwelling on cliffs, as in kittiwakes and other gull species, involves a different reaction to depth compared with ground-nesting gull species [3–5]. Evaluation and reaction to depth involves depth perception and discrimination and is fundamental for survival [6]. Following the occupation of different habitats, even related species display different behavioral traits that enable them to maximize their fitness and survival.

**Fig 1:**
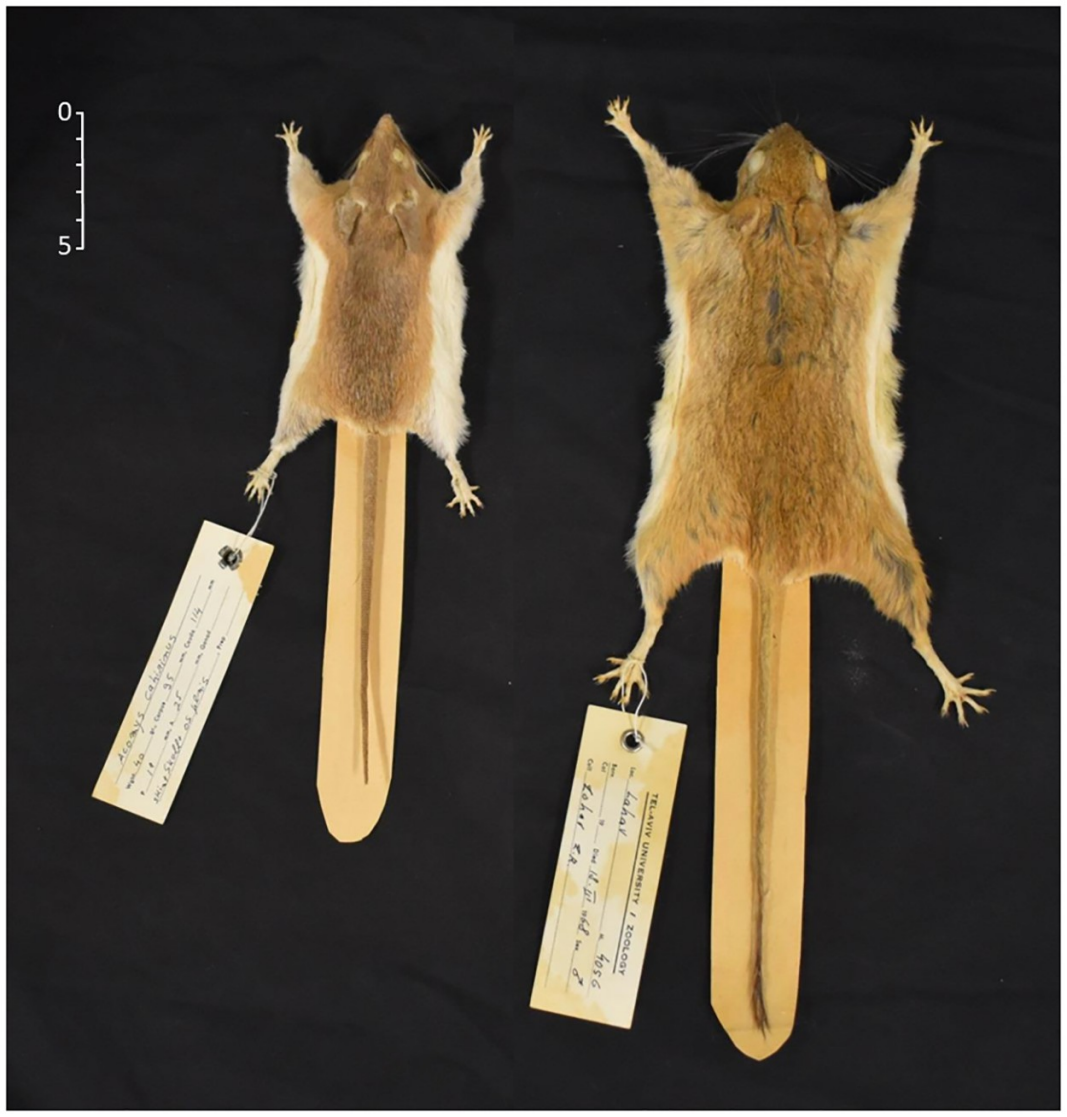
The anatomical difference between common spiny mouse (left) and Tristram’s jird (right). As shown, the hindlegs in the jird are about 1.5-fold longer than its forelegs; while in the spiny mouse foreleg and hindleg length are almost the same [courtesy of Steinhardt Museum of Natural History, at Tel-Aviv University].

The adaptations that animals undergo in natural habitats are traits that can also be discerned when these animals are tested in a relatively simple and impoverished laboratory environment. Moreover, testing different species from different natural habitats in the same simple laboratory environment can uncover the specific abilities and adaptations that differentiate these species according to their natural habitats. In the present study, we used a three-dimensional laboratory environment to test two rodent species: Tristram’s jirds (*Meriones tristrami*), which live in flatlands and prairies [7–9]; and common spiny mice (*Acomys dimidiatus*), which live in a rocky complex habitat [9,10]. Another factor that influences spatial behavior in a specific habitat is that of motor ability. Different species travel using gaits appropriate to their anatomy and habitat. Indeed, jirds run using ’bound’, a fast gait based on leaping on their relatively long hindlegs and landing on their shorter forelegs after a relatively long aerial phase—a gait that is effective in flatlands [11,12]. Spiny mice, which compared to jirds have relatively short hindlegs (Fig 1), progress in a sequence of brief leaps with frequent but short aerial phases, a mode of progression that is more appropriate when jumping from one rock to another [13]. In the present study, the spatial behavior of jirds and spiny mice was compared in order to determine how their adaptations to different habitats are manifested in their spatial behavior when they explore a three-dimensional environment.

When a rodent is introduced into an unfamiliar environment, its behavior constitutes a set of roundtrips (excursions) to a specific location—the home-base [14]. Specifically, the rodent stays at the home-base for extended periods, pays the highest number of visits to it and usually crouches there. The home-base is a terminal for roundtrips of exploration, with a slow and intermittent outbound section and fast direct inbound section [15]. Numerous studies have described exploration and home-base behavior in several species [14,16,17], and its controlling mechanisms [18]. Rodents set their home-base near a salient landmark [19], which in an empty arena (open-field) is usually in one of the corners, perhaps due to the sense of safety [20,21]. In more complex environments, it was found that rats tend to set their home-base at a vantage point [22]. The majority of studies on spatial behavior in rodents was performed in two-dimensional laboratory environments. In real life, however, rodents and other animals confront various types of three-dimensional environments in their natural habitats, such as slope terrain, multi-level environments and orthogonal planes [23]. The question posed in this study was that of how jirds and spiny mice explore and travel in a three-dimensional environment, considering their different natural habitats and modes of progression.

## Materials and methods

### Animals

Ten male Tristram’s jirds (*Meriones tristrami*; body length: 136 ±13mm; tail length: 135 ±10mm; weight: 70±14g; [9]) and nine female and male common spiny mice (*Acomys dimidiatus*; previously considered the subspecies *A*. *cahirinus dimidiatus*; body length: 114±8; tail length: 97±22mm; weight: 41±9 g; [9]), were obtained from captive colonies at Tel-Aviv University. All rodents were adults (3- to 12-month-old). The zoo colonies are kept under natural light and temperature conditions, in a 120 x 60 x 60 cm cages, in each 5–20 males and females. These cages include boxes, ceramic pots, and wire mesh walls on which the rodent like to climb. The rodents were housed in a temperature-controlled room (23 ± 1°C) for one day before testing in rodent cages (42 x 26.5 x 18.5 cm) with sawdust bedding (1–3 animals per cage). Standard rodent chow and fresh water were provided ad-lib.

#### Ethics approval

This study was carried out in strict accordance with the regulations and recommendations of the Institutional Committee for Animal Experimentation at Tel-Aviv University. This committee approved the protocol of this study (permit 04-19-066). Notably, this was a non-invasive study, which involved no harm, pain or distress to the animals.

### Apparatus

**Fig 2:**
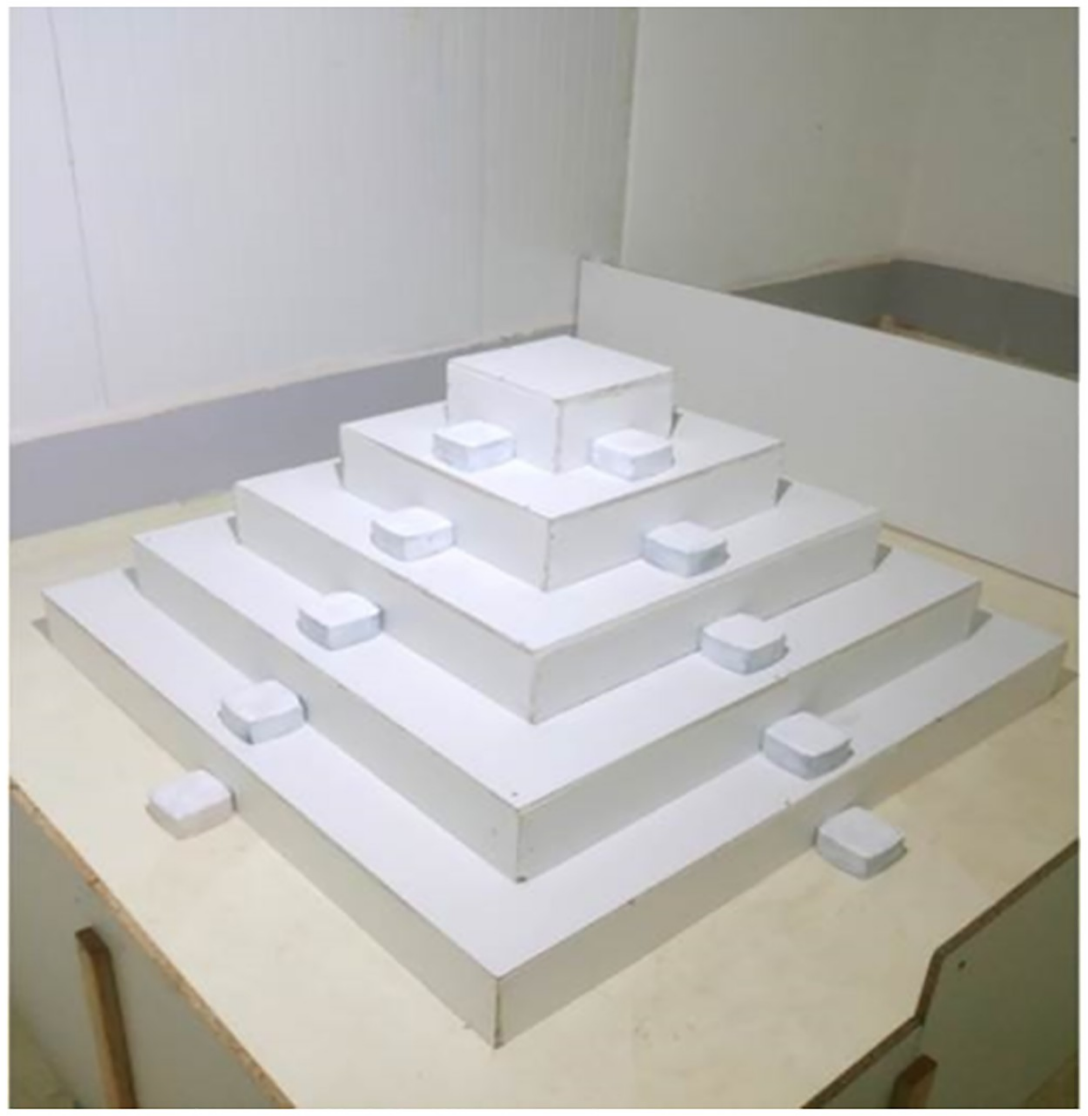
The ziggurat apparatus (five-level pyramid).

A 250 x 250 cm arena with 50 cm high walls was located in a light-proofed 6 x 6 m air-conditioned room (23°C). A plywood ziggurat (pyramid) was placed in the center of the arena and comprised five levels: 150 x 150 cm, 120 x 120 cm, 90 x 90 cm, 60 x 60 cm and 30 x 30 cm, each 15 cm high. A concrete brick (11 x 11 x 5 cm) was placed in the center of each side at each level of the pyramid to provide the rodents with easy-to-climb locations (Fig 2). A video camera (Minitron MTV-73S85H color CCTV) was placed 2.5 meters above the center of the ziggurat, capturing the entire arena. The apparatus was illuminated with dim light (1.31 lux). An IR light (Tracksys, UK) invisible to the rodents was also used to illuminate the arena in order to provide a vivid image for the tracking system. The image of the rodent in the video signal was tracked by *EthoVision XT* 11.5 (by *Noldus Information Technologies*, NL; [24] at a rate of 25 frames per second.

### Procedure

After turning on the camera and the tracking system, an individual rodent was gently released from a jar onto the middle of the second level of the pyramid. The experimenter then left the room and videotaping continued for 60 min. At the end of the session the rodent was returned to its cage and the arena was mopped with soap and water to neutralize odors. All sessions took place during light hours (8 am-8 pm).

### Data acquisition and analysis

X, Y, T coordinates were extracted automatically from EthoVision at a rate of 25 frames/sec and transferred to R software (version 4.0.2; [25]) to calculate the following parameters:

**Distance travelled on the floor and on the pyramid**. The metric distance travelled on the floor and on the levels of the pyramid as calculated by R from the X-Y coordinates. In addition to the total travelled distance (floor and pyramid), the distance was adjusted to the respective areas of each (distance travelled on the pyramid divided by pyramid area, and distance travelled on the floor divided by floor area).
**Cumulative time spent on the floor and on the pyramid**. The cumulative time spent on the floor and on the levels of the pyramid as calculated by R from the X-Y-T coordinates. The time on the floor and the pyramid was adjusted according to the area, as described for the distance above.
**Temporal order of the first arrival on the floor and at each of the pyramid levels**. The number of rodents reaching each of these sectors for the first time was scored at 10-min intervals.
**Moving between levels**. The total number of episodes of moving from one level to the next was calculated for each rodent. The proportion of switching between each two adjacent levels was then calculated out of the total number of switching episodes.
**Key stopping locations**. X,Y,T coordinates of the stops were fed into SEE software [26–28], and a stop was considered as the animal remaining at the same location for at least three seconds [29]. The set of these stopping locations was fed into a custom R version of the City Clustering Algorithm ([30,31]; for the application of the algorithm in the study of spatial behavior, see [32]) and clusters with at least three stops within a diameter of body length (13.5 cm for jirds and 11.5 cm for spiny mice; [9]) were considered as key locations.
**Home-base location**. For each rodent, key locations were ranked according to the cumulative time spent there. The top-ranked location, where the rodent spent the longest duration, was taken as its home-base if it was statistically greater from the second rank (one-way ANOVA followed by a Fisher’s LSD post-hoc test). If the second-ranked location differed from the third-ranked location, it was defined as a secondary base.
**All data of the above parameters are available in Supporting information** S1 Data.

### Statistics

Unless noted otherwise, one- or two-way analysis of variance with repeated measures was performed and followed by Fisher’s LSD post-hoc test. Data that deviated from normal distribution were adjusted by square-root transformation. Alpha level was set to 0.05 in all tests.

## Results

**Fig 3:**
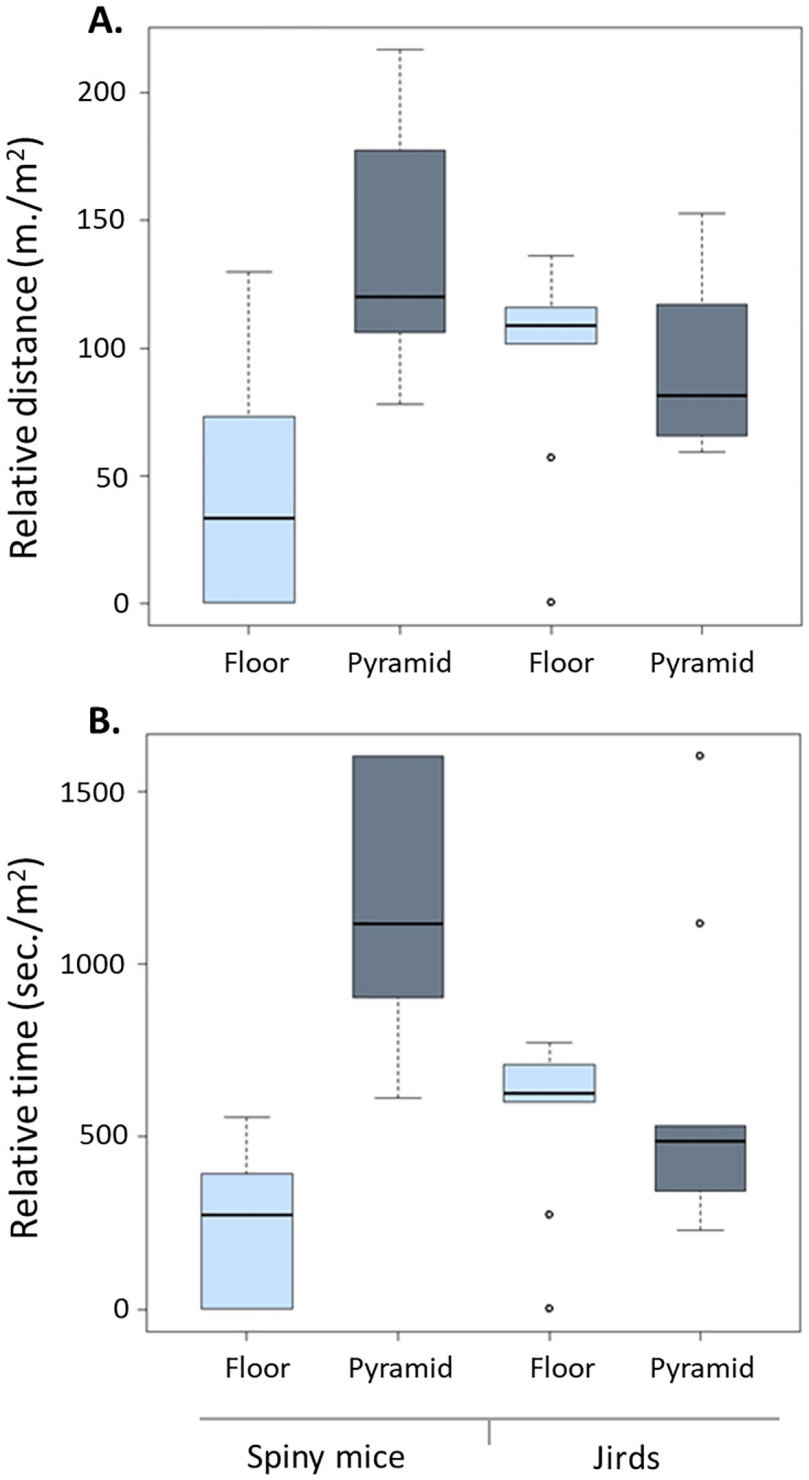
Box plots of the distance travelled per square meter (A) and the time spent per square meter (B) on the floor *vs*. the pyramid by spiny mice and by jirds. As shown, the two species displayed a different division of activity between the floor and the pyramid. A Fisher’s LSD post-hoc test revealed that in both distance travelled and time spent, there was a significant difference between floor and pyramid in spiny mice but not in jirds.

The total distance travelled (on the floor and pyramid together) during the 60 min test did not differ between jirds and spiny mice (mean ± SEM: 593 ± 125 m vs. 472 ± 245 m for jirds and spiny mice, respectively). To compare the distances travelled and the time spent by each rodent on the floor and on the pyramid, we adjusted to the measured distance and measured time on each to the respective areas of the floor and of the pyramid. We found that jirds travelled about the same distance on the floor and on the pyramid (mean ± SEM: 98 ± 14 and 92 ± 11 m/m^2^, respectively), and similarly spent about the same time on the floor and on the pyramid (mean ± SEM: 566 ± 81 and 595 ± 144 sec/m^2^, respectively). In contrast, spiny mice travelled a greater distance on the pyramid (mean ± SEM: 138 ± 17 m/m^2^ on the pyramid and 42 ± 17 m/m^2^ on the floor) and spent more time on it than on the floor (mean ± SEM: 1210 ± 142 sec/m^2^ on the pyramid and 221 ± 80 sec/m^2^ on the floor; Fig 3). Indeed, a two-way analysis of variance with repeated measures between the distance travelled on the floor and on the pyramid by the two species revealed no difference between species (F_1,17_ = 0.16, p = 0.6970), a significant difference between floor and pyramid (F_1,17_ = 9.41, p = 0.0069), and a significant interaction between species and floor/pyramid (F_1,17_ = 12.06, p = 0.0029). Applying the same comparison for the time spent on the floor and on the pyramid revealed difference between species (F_1,17_ = 10.26, p = 0.0052), difference between floor and pyramid time (F_1,17_ = 11.52, p = 0.0035), and a significant interaction between species and floor/pyramid time (F_1,17_ = 10.25, p = 0.0052). Altogether the jirds and spiny mice revealed a different division of their activity between the floor and the pyramid.

**Fig 4:**
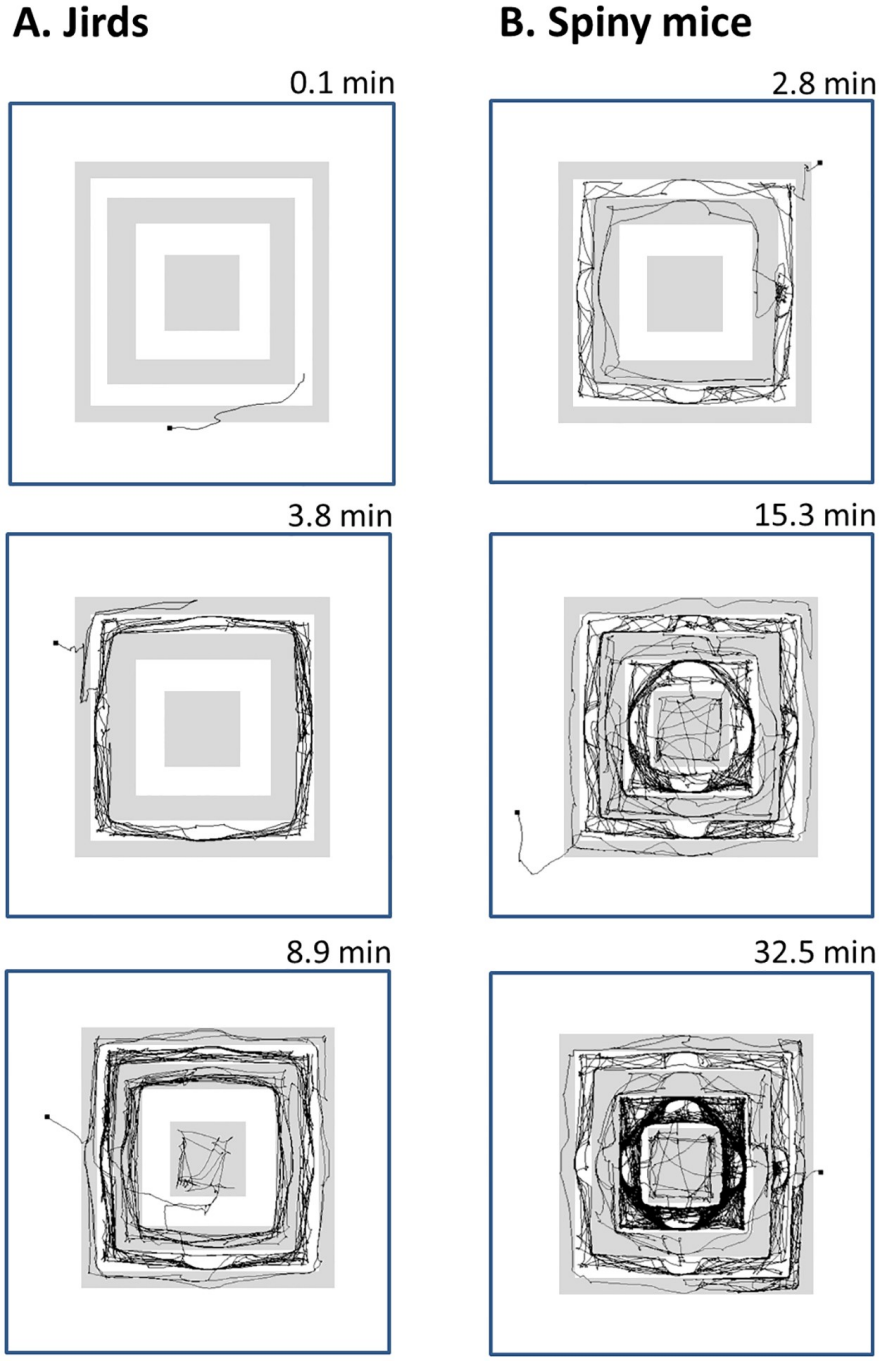
Trajectories of the routes passed by jirds (A) and spiny mice (B) are depicted for the period from the start until the first arrival to the floor. The jird and the spiny mouse that were fastest to reach the floor are depicted at the top row. The jird and spiny mouse that were closest to the mean time of the group for reaching the floor are depicted in the middle row, and the jird and spiny mouse that were latest to reach the floor are depicted at the bottom row (the five spiny mice that never reached the floor were excluded from this figure). As shown, jirds preferred to soon descend down to the lower levels and the floor. The jird at the bottom row was the only one that ascended above the starting level before descending down. In contrast, spiny mice travelled the pyramid levels for longer time (and distance), and all of them climbed above the starting level before descending to lower levels or to the floor.

Following introduction onto the second level of the pyramid at the beginning of the test, nine out of the 10 jirds visited the two bottom levels and floor during the first 10 minutes, and reached the third floor, which was above the start point, only after 20 minutes. In contrast, seven out of the nine spiny mice visited the highest, fifth, level already in the first 10 minutes, and four of them never descended to the floor throughout the entire 60 min test. This preference by jirds to descend the pyramid was also manifested in their earlier first arrival on the floor compared with spiny mice (mean ± SEM: 3.1 ± 0.8 min and 15.5 ± 6.4 min for jirds and spiny mice, respectively; one-way analysis of variance ANOVA, F_1,12_ = 6.71, p = 0.024). Trajectories of the routes passed until the first arrival to the floor are depicted for the jirds and spiny mice that were the first, average, and latest to reach down to the floor (Fig 4).

**Fig 5:**
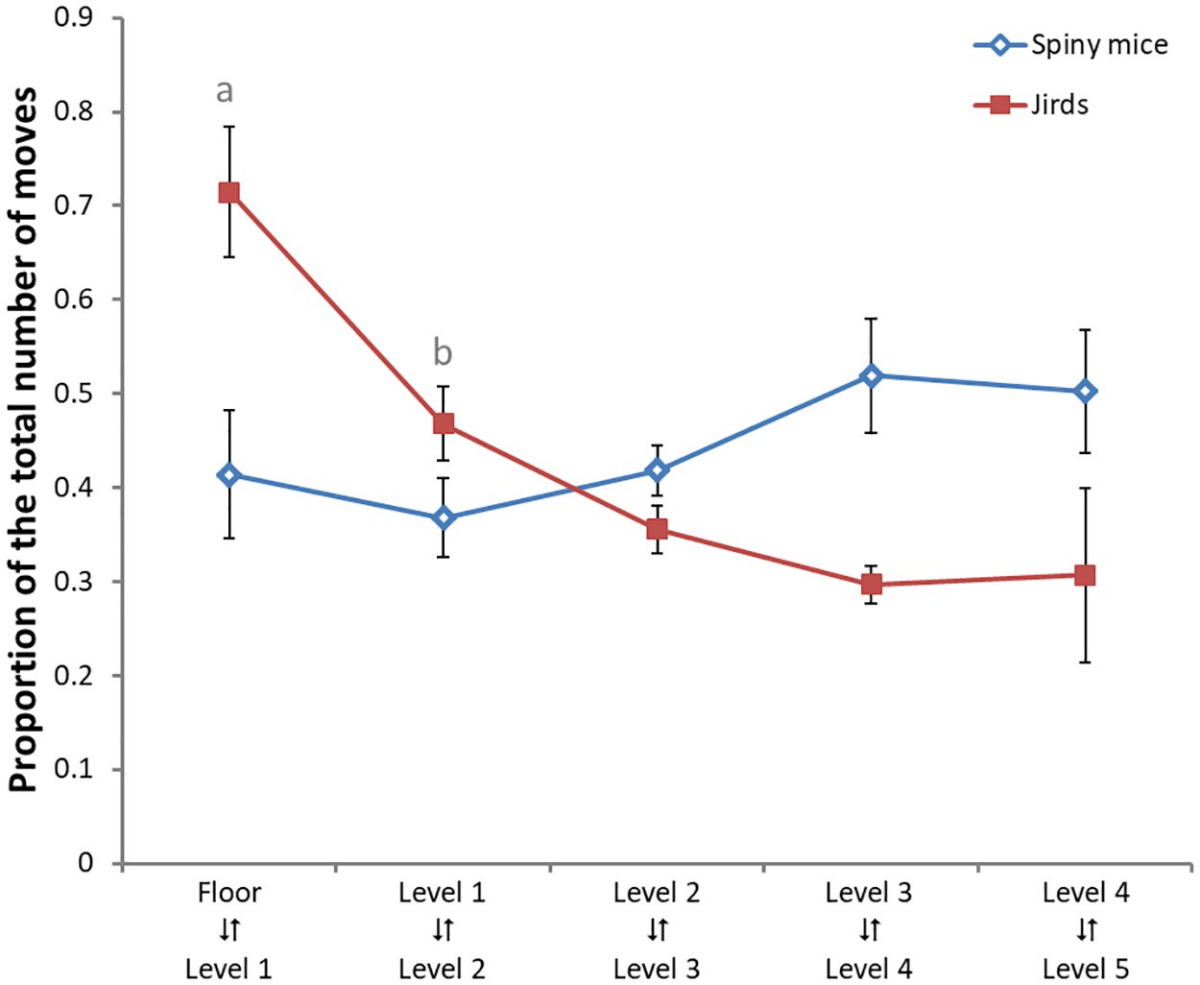
Transitions between the pyramid levels. Pairs of two adjacent pyramid levels are depicted along the abscissa, and the mean proportion (± SEM) of transitions between each pair of adjacent levels out of the total number of transitions during the 60 min test is depicted along the ordinate as proportion. As shown, the number of tansitions between two adjacent levels in jirds declined with their progress to the pyramid top; while in spiny mice it remained steady over all levels. A Fisher LSD post-hoc test revealed that jirds moved between the floor and the lowest pyramid level (level 1) significantly more than between all other levels (a), and the proportion of transitions between levels 1 and 2 were greater than between levels 3 and 4 (b). In spiny mice there was no significant difference in the proportion of transitions between the different levels and they indifferently moved between the pyramid levels.

Jirds also differed from spiny mice in moving between the apparatus levels. Specifically, the higher the level, the less the jirds ascended or descended to or from it (mean ± SEM number of transitions between levels: Floor ↔ Level 1, 56 ± 8; Level 1 ↔ Level 2, 27 ± 5; Level 2 ↔ Level 3, 17 ± 4; Level 3 ↔ Level 4, 11 ± 3; Level 4 ↔ Level 5, 10 ± 4). In contrast, spiny mice equally ascended and descended among the various levels (mean ± SEM number of transitions between levels: Floor ↔ Level 1, 34 ± 15; Level 1 ↔ Level 2, 22 ± 9; Level 2 ↔ Level 3, 21 ± 7; Level 3 ↔ Level 4, 21 ± 5; Level 4 ↔ Level 5, 17 ± 4). There was an individual variability in this measure since some jirds did not ascend all the pyramid levels and some spiny mice did not descend to the floor, we therefore calculated the proportion of transitions between levels, as depicted them in Fig 5. Two-way analysis of variance with repeated-measure ANOVA revealed no difference in the number of level transitions between species (F_1,17_ = 3.20, p = 0.091), a significant difference between levels (F_4,68_ = 2.71, p = 0.037) and an interaction between species and transitions (F_4,68_ = 6.16, p < 0.001).

**Fig 6:**
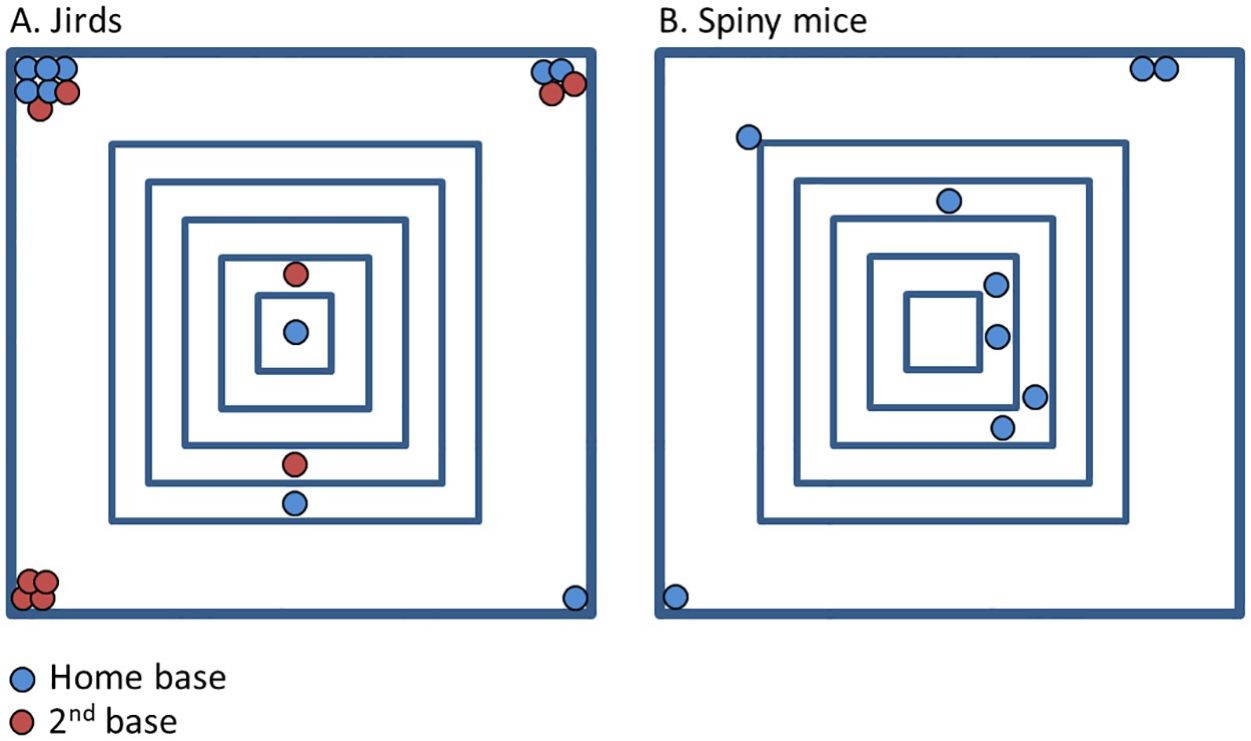
The location of home-bases and secondary bases in jirds (A) and spiny mice (B). As shown, jirds established eight home-bases on the arena floor (blue circles) and two home-bases on the pyramid. In addition, they establish eight second-bases (red circles) on the arena floor and two on the pyramid. Spiny mice (B) established five home-bases on the pyramid, one home-base on the floor next to the pyramid, and three home-bases on the arena floor (spiny mice did not establish secondary bases).

As detailed in the ’Methods’ section, key stopping locations were those that were visited for three or more seconds at least three times. Ranking the 11 key locations of jirds from high to low according to the cumulative time spent in each location revealed that jirds spent significantly more time in the two top ranks than in any other location (one-way analysis of variance ANOVA; F_10,98_ = 26.26, p < 0.001; 1^st^ ranked location 10.5 ± 1.6 min and 2^nd^ ranked location 7.0 ± 1.1 min on average; difference between ranks was revealed in a Fisher’s LSD post-hoc). We therefore considered the first key location as the home-base and the second key location as a secondary base. Notably, while the home base and the second base of jirds were mostly at the arena corners, the jirds did not necessarily spent extended periods in the other arena corners. Indeed, two jirds established their bases on the pyramid, with none of the arena corners being among their four top-ranked locations. In other jirds the third and/or fourth ranked locations were not necessarily corners. In spiny mice, only the first-rank key location (out of 18 key locations) was significantly greater than all other locations in terms of the time spent in it (one-way analysis of variance ANOVA; F_17,144_ = 5.12, p < 0.001; 1^st^ ranked location 9.5 ± 3.8 min and 2^nd^ ranked location 4.3 ± 2.0 min on average; difference between ranks was revealed in a Fisher’s LSD post-hoc; see Supporting information S1 Data), and was therefore considered as the spiny mice home-base. The various locations of home-bases and secondary bases are depicted in Fig 6. As shown, spiny mice established their home-bases mainly on the pyramid, whereas jirds favored locations on the floor as their home-bases and secondary bases.

**Fig 7:**
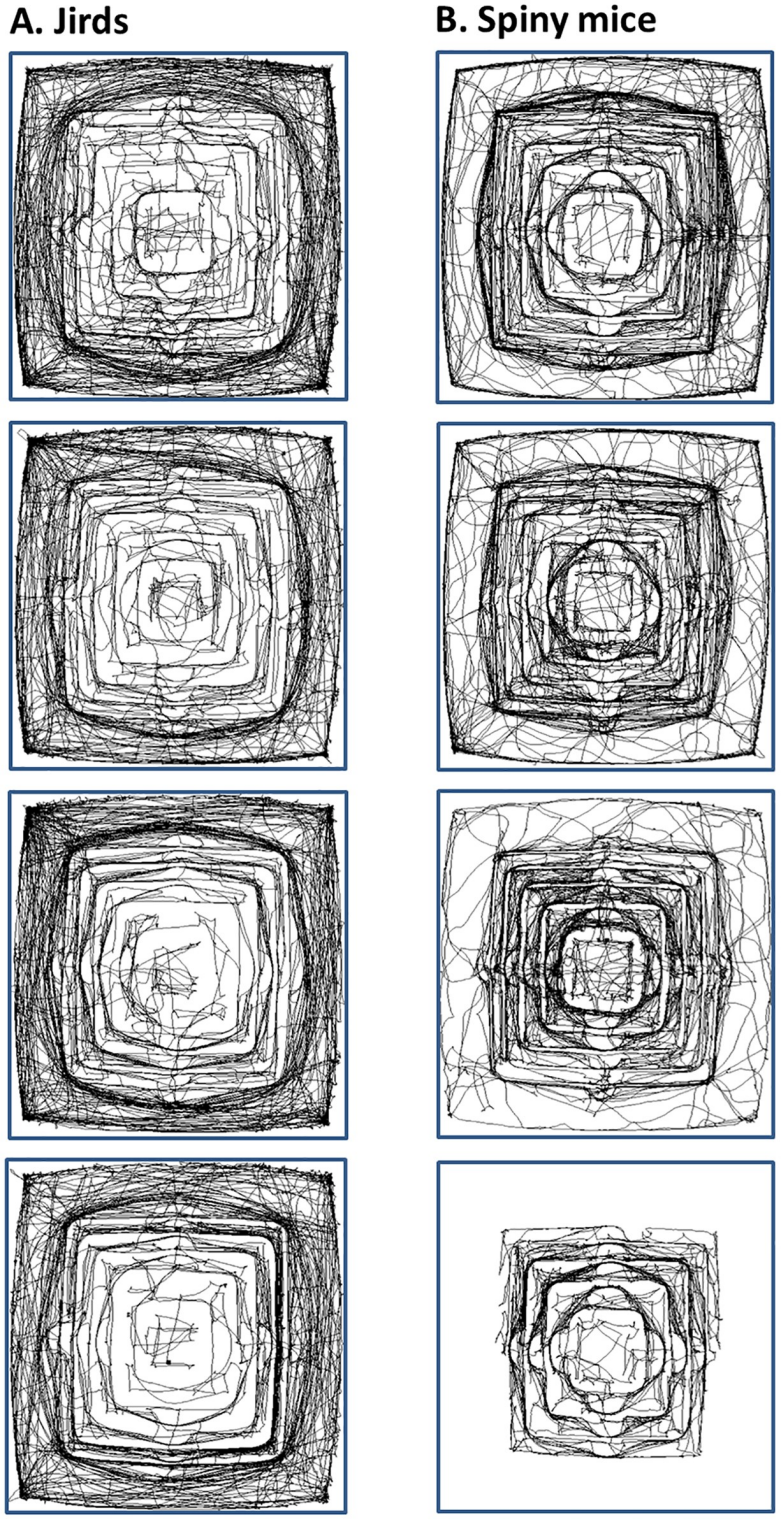
Representative trajectories of locomotion of jirds (A) and spiny mice (B). As shown, jirds travelled mostly on the floor or near it, whereas spiny mice travelled mostly on the pyramid and next to its base (note that in the figure there is one spiny mouse that never descended to the floor; another three spiny mice also did not get to the floor). Note that the structure of the apparatus is apparent solely from the trajectories.

Fig 7 depicts the trajectories of four jirds and four spiny mice, demonstrating that jirds travelled mostly on the floor whereas spiny mice travelled mostly on the pyramid or next to its base on the floor. This reflects Fig 3, reconfirming the preference of jirds to travel on the floor or near it and establishing their bases there, in contrast to the spiny mice’s preference to travel on the pyramid and establish their home-bases there. On the pyramid, jirds mainly travelled on the lower levels whereas spiny mice travelled equally on all the levels.

## Discussion

Surface-bounded animals necessarily encounter various three-dimensional structures in their living habitat. Here we compared the spatial behavior of jirds, which dwell in flatlands, with that of spiny mice, which dwell in structured rocky habitats. Individuals of these two species were each introduced separately onto a five-level ziggurat (pyramid) located in the center of a large arena. As hypothesized, the spatial behavior of both rodent species reflected their natural habitat and motor abilities, with jirds favoring travel on the floor or lower pyramid levels, and spiny mice favoring travel on the pyramid. In the following, we discuss these results as a reflection of differences in affordance and *umvelt*, as well as a possible difference in depth perception.

Jirds and spiny mice occupy different habitats and were therefore selected for testing in order to uncover possible differences in their three-dimensional spatial behavior. The typical rodents spatiotemporal behavior—of establishing a home-base and setting out from it to explore the environment [14]—was evident in both the jirds and spiny mice in the three-dimensional environment of the present study. This is in agree with the documentation of home-base behavior in other studies in a variety of three-dimensional apparatuses [22,33,34]. The behavior of the two species differed, however, in the location of home-base: spiny mice typically established their home-bases on or near the pyramid, whereas jirds tend to establish the bases on the floor corners. This difference in home-base location is noteworthy, since rodents tend to establish a home-base near a salient landmark [19,35]. Here it seems that for spiny mice, the salient landmark was the pyramid whereas for jirds salient landmarks were the arena corners. It seems that each species e running gaits are more efficient in flatlands while jumping is more suitable for a rocky habitat employed different considerations regarding home-base location. Jirds are burrow-dwellers and, therefore, we assume that by establishing their home-bases mostly on the floor, they were seeking safety [20]; whereas spiny mice, which dwell in crevices between rocks and boulders, were seeking a location that could be used as a vantage point [22]. This difference was echoed in the preference of jirds to travel mainly on the floor or near it, in contrast to that of the spiny mice to travel on the pyramid. Thus, it would appear that the floor was the attractor for spatial behavior in the jirds, whereas the spiny mice were attracted to the pyramid, with some of them (4 out of 9) never reaching the floor.

Spiny mice dwell and forage in the crevices among boulders and rocks [9,10]. In this habitat, they are relatively protected from predators, such as owls and foxes [36–38]. Accordingly, in the test arena they restricted their travel to the walls of the pyramid, and avoided the relatively open area of the floor. The spiny mice were clearly not attracted to the peripheral arena walls but to the complex three-dimension ziggurat (Fig 7), which more closely resembled their natural habitat. The jirds, in contrast, dwell in flatlands and prairies [7–9] in either vegetated or open patches [7] and accordingly, they mainly explored the lower, more exposed parts of the environment (Fig 7).

Jirds and spiny mice dwell in different habitats in which they travel using different gaits that are adapted to their anatomy and habitat. In jirds, the hindlegs are about 1.5-fold longer than the forelegs (Fig 1) and they run in a gait termed ’bound’ [11,12], which is typical to small mammals [39]. Bounding is based on leaping simultaneously on the long hindlegs and landing simultaneously on the shorter forelegs, with a relatively long suspension phase between the lift-off of the hindlegs and the touch-down of the forelegs [40]. Accordingly, in the suspension phase of this gait, the airborne trajectory of moving up against gravity is shorter than that of moving down with gravity. Accordingly, there is an energetic restoration due to the longer downward movement [11], making this gait efficient for animals that live in flatlands. Such a gait, however, is not suitable for a structured habitat in which continuous ascent and descent is required during progression. Indeed, spiny mice that live in such a habitat progress by means of brief and short leaps with a short suspension phase between hindleg and foreleg stepping (primitive ricochet; [41]), much like Asian garden dormice (*Eliomys melanurus*) that also live among rocks and boulders [12]. Spiny mice are also known for their jumping ability in both the vertical and horizontal domains [42]. Thus, running gaits are more efficient in flatlands while jumping is more suitable for a rocky habitat [42]. Therefore, the preference found here of the jirds for the floor and the spiny mice for the pyramid would seem to suit their different modes of progression. While both jirds and spiny mice displayed the same spatiotemporal structure of rodent exploration, which is organized in reference to a home-base, their differential spatial behavior in a three-dimensional environment, therefore, reflects the features of their natural habitat and motor abilities.

Travelling in a three-dimensional environment requires height and depth perception. Studies on height perception in humans have found that spatial perception is distorted by non-optical factors such as mood [43] and the physical effort required when ascending or descending [44]. There is also a tendency to overestimate height and slopes when viewed from the top [45]. Estimating height depends, among others, on the reaction to depth and the ability to correctly perceive depth. Jirds are an altricial species in which the offspring depend on parental care for several weeks after birth [46], whereas spiny mice are precocial and their offspring can become independent within a few days postnatally [47]. Previous studies have revealed differences in depth perception between precocial and altricial species [6,48–50]. Generally, precocial newborns are able to discriminate depth without prior experience [48,51], whereas in altricial species individuals without prior experience have less accurate depth perception [48]. However, there are some altricial mammals (e.g. laboratory rat pup, *Rattus norvegicus*; kitten, *Felis silvestris catus*; rhesus monkey infant, *Macaca mulatta*) that acquire a good discrimination of depth without much prior experience [48,49]. Nevertheless, studies in young animals have revealed that experience and practice in complex environments, including cliffs, lead to better discrimination of depth [50,52,53]. Tristram’s jirds, in a three-dimensional environment surrounded by cliffs, prefer to stay in the center away from the cliffs, whereas sand rats (*Psamomys obesus*), which forage by climbing on shrubs, prefer the perimeter near the cliffs [33]. The Mongolian gerbils (*Meriones unguiculatus*), a species that is quite similar in morphology and habitat to the Tristram’s jirds of the present study, was found to possess a relatively poor depth perception [54], and this seems to also occur in Tristram’s jirds that avoided cliffs [33], in accordance with their flat natural habitat. In contrast to jirds, testing another species of common spiny mice (*Acomys cahirinus*; which is very close to the *A*. *dimidiatus* of the present study) on a visual cliff and under platforms of different heights, revealed that they possess a good depth-perception even within a few hours after birth; and that before jumping they adjust their posture precisely to achieve the required distance and height [42]. In light of the above studies, it is suggested that the present finding that the precocial spiny mice mostly travelled on the pyramid while the altricial jirds mostly travelled on the floor, seems to indicate a better depth perception by the former compared to the latter.

*Umvelt* is a term pertaining to the different animals’ perceptual worlds [55]. According to this notion, the same physical environment is differently perceived by different animals since each species focuses on different environmental attributes. A complementary notion is that of *affordance*, which was introduced by Gibson [56], suggesting that each species exploits differently the same attributes of the physical environment. These two complementary notions of *Umvelt* and *Affordance* seem to offer an effective explanation for the results of the present study: two species, each dwelling in a different habitat, have different motor abilities, and probably also different depth perception. Consequently, they distribute their activity differently over the same three-dimensional physical environment while maintaining the typical home-base behavior of rodent exploration.

### Conclusion

Rodents demonstrate a typical structure of exploring an unfamiliar environment: they establish a home-base, which is a location in which the stay for extended periods, crouch, and display extended bouts of grooming, and from which they set out on roundtrips to explore the environment. This spatiotemporal structure of this behavior is shown to be preserved here in two rodent species, each dwelling in a differently structured environment. The two species, however, differed in the distribution of their spatial behavior in a three-dimensional test environment. Jirds, which live and forage in flatlands, split their travel and time equally between the floor and the lower levels of the pyramid, yet favored to establish their bases in the arena corners. In contrast, activity of spiny mice, which live and forage in the crevices between rocks and boulders, was mostly bounded to a pyramid located in the center of the arena. Notably, jirds are about twice the length and mass of spiny mice and this difference could partially account for some of the above differences. Studying more species is therefore desired to further generalize the present results. However, the distinctly different distribution of activity in the same three-dimensional environment reflects the different motor abilities and depth perception that were previously described in spiny mice and accord with the adaptations required for animals dwelling in vertically structured habitats compared to those from flatlands.

## Supporting information

S1 Data Data of all results. The attached Excel file provides the raw data of the results and the respective statistics. Each spreadsheet includes the data for the variable specified in its title.
(XLSX)
